# Enhanced mnemonic discrimination for emotional memories: the role of arousal in interference resolution

**DOI:** 10.3758/s13421-020-01035-3

**Published:** 2020-04-20

**Authors:** Ágnes Szőllősi, Mihály Racsmány

**Affiliations:** 1 Department of Cognitive Science, Budapest University of Technology and Economics, Egry Jozsef utca 1, Budapest, 1111 Hungary; 2 Institute of Cognitive Neuroscience and Psychology, Research Centre for Natural Sciences, Budapest, Hungary

**Keywords:** Emotion, Arousal, Interference, Pattern separation, Hippocampus

## Abstract

In the present study we investigated the long-standing question whether and why emotionally arousing memories are more distinct as compared to neutral experiences. We assumed that memory benefits from the distinctiveness of emotional information, and that emotions affect encoding by reducing interference among overlapping memory representations. Since pattern separation is the process which minimizes interference between memory representations with similar features, we examined the behavioral manifestation of putative neural mechanisms enabling pattern separation (i.e. mnemonic discrimination) for emotionally arousing materials using the Mnemonic Similarity Task with negative, positive, and neutral images as stimuli. Immediately after incidental encoding, subjects were presented with stimuli they had seen at encoding and also with new items. Crucially, participants were also presented with lure images that were visually similar to ones they had seen before. Response options were old, new, and similar. Our results showed that individuals were better in discriminating between similar, emotionally arousing memories, when compared to the neutral stimuli. Moreover, this so-called lure discrimination performance was better for the negative images, than it was for the positive stimuli. Finally, we showed that the high arousing negative stimuli were better separated than the low arousing negative stimuli, and a similar pattern of results was found for the positive items. Altogether, these findings suggest that lure discrimination is modulated by arousal and not by valence. We argue that noradrenergic activity might facilitate interference resolution among memory representations with similar features, and that superior pattern separation might play a key role in memory enhancement for emotional experiences.

## Introduction

It has long been demonstrated that emotional memories are better remembered (for reviews, see Hamann, 2001; Kensinger, 2009; Labar & Cabeza, 2006; McGaugh, 2015; Phelps, 2004; Talmi, 2013) and are more accurate (Heuer & Reisberg, 1990; Kensinger, 2009; but see Rimmele, Davachi, Petrov, Dougal, & Phelps, 2011), than memories for neutral experiences. Additionally, emotional memories are more vivid (Heuer & Reisberg, 1990; Kensinger & Corkin, 2003; Todd, Talmi, Schmitz, Susskind, & Anderson, 2012) and are recollectively re-experienced when accessed as compared to neutral experiences (which are associated with fewer “Remember” responses), indicating that emotions promote episodic remembering (Dewhurst & Parry, 2000; Kensinger & Corkin, 2003; Rimmele et al., 2011; for an overview, see Yonelinas & Ritchey, 2015). For example, Dewhurst and Parry (2000) found a bias toward “Remember” responses for emotional words (as compared to the ratio of Know responses) which suggests recollective remembering for emotional words. Kensinger and Corkin (2003) reported a similar pattern of findings and showed that this effect was more pronounced for stimuli that evoked arousal as compared to valenced but non-arousing stimuli. In brief, this emotional memory enhancement specifically characterizes memory for arousing information, rather than memory for non-arousing stimuli (see also Cahill & McGaugh, 1995; Kensinger & Corkin, 2003, 2004).

In the present study we aimed to further investigate memory for emotionally arousing materials. Recently, it was suggested that a process, called pattern separation, is a key aspect of episodic remembering (see Yassa & Stark, 2011 for a detailed overview). Pattern separation is a computational mechanism that allows the reduction of interference effects between overlapping memory representations, and therefore allows the encoding and storage of (episodic) memories for unique events together with their contextual features. We believe that this process plays a crucial role in memory enhancement for arousing information, therefore, we designed a study to examine interference resolution and the formation of distinct (unique) memories for arousing materials.

### Emotion, arousal, and memory

Several theorists highlight the role of arousal in emotional memory enhancement. For example, based on the results of early animal studies, McGaugh (2000) proposed that arousal modulates long-term memory consolidation. Accordingly, most human studies found improved memory for arousing materials after relatively long delays of days or even weeks, but not when retrieval occurred immediately or shortly after learning (Hamann, Ely, Grafton, & Kilts, 1999; Sharot & Yonelinas, 2008). According to this consolidation framework, arousing experiences trigger the secretion of stress hormones (an increase in noradrenergic activity and an increase in glucocorticoid levels) that interact with the amygdala and affect long-term memory retention (see also McGaugh, 2002, 2004, 2015).

Supporting this assumption (McGaugh, 2000), the amygdala shows greater activity for emotionally arousing materials at encoding, and this increase in amygdala activity is associated with better subsequent long-term, but not immediate, memory performance (Cahill et al., 1996; Cahill, Uncapher, Kilpatrick, Alkire, & Turner, 2004; Hamann et al., 1999; Sommer, Gläscher, Moritz, & Büchel, 2008; Tabert et al., 2001). During successful encoding of emotionally arousing study materials, the activations of the amygdala and the hippocampus correlate (Hamann et al., 1999). While the hippocampus is always activated at encoding, the amygdala responds only to emotionally arousing stimuli. Moreover, it has been suggested that the amygdala-hippocampal network regulates memory enhancement for arousing information but not memory for valenced but non-arousing stimuli (see Kensinger & Corkin, 2003, 2004).

Within such a consolidation framework, however, some results remain difficult to explain. It has been demonstrated that emotional arousal improved performance when memory was tested immediately or a few minutes after encoding (Chainay, Michael, Vert-pré, Landré, & Plasson, 2012; Dewhurst & Parry, 2000; Schmidt & Saari, 2007; but see e.g. Christianson & Nilsson, 1984), and that increased amygdala activity during encoding predicted immediate memory performance (Hamann & Mao, 2001). Several theorists stressed that arousal affects encoding as an indirect consequence of enhanced attention. That is, since arousal enhances attention, it leads to a more elaborative encoding of arousing information (see Hamann, 2001; Talmi, Schimmack, Paterson, & Moscovitch, 2007). Others highlighted that memory benefits from emotions due to the distinctiveness of arousing stimuli that leads to differential processing of information, such as post-stimulus elaboration and increased individual item processing (Schmidt & Saari, 2007; Talmi, 2013).

To summarize, for successful remembering, three phases must occur successfully, as reviewed by Kensinger (2009). First, the event must be encoded. It has been shown that arousing information is more likely to be attended (e.g. Talmi et al., 2007) and the encoding of such information is highly elaborative as compared to the encoding of non-arousing information (e.g. Schmidt & Saari, 2007; for overviews, see Kensinger, 2004; Talmi, 2013). Second, a long line of studies showed better memory for arousing information following relatively long delays of days or even weeks (e.g. Hamann et al., 1999; Sharot & Yonelinas, 2008) indicating that arousal has a beneficial effect on memory consolidation processes. In other words, it seems that once the arousing information is encoded, it tends to persist in the long term (see McGaugh, 2000, 2015). Finally, following the (elaborative) encoding and (successful) consolidation of an information, the event must be accessed during retrieval. There has been a growing body of evidence that arousal exerts effects during retrieval (e.g. Dolan, Lane, Chua, & Fletcher, 2000; for an overview, see Buchanan, 2007), however, how emotions and arousal affect search processes at retrieval is still under debate.

Two other aspects of memory for emotional information are especially important from the point of view of our study. *First*, a distinction should be made between the arousal level of a stimulus and its emotional valence (positive or negative). Beyond the role of arousal, interestingly though, only a relatively few studies investigated memory for valenced but non-arousing stimuli. However, it has been demonstrated that memory for such materials is better, when compared to memory for non-valenced, non-arousing stimuli (Kensinger & Corkin, 2003; Ochsner, 2000). It has also been shown that negative memories are better remembered when compared to positive memories (Ochsner, 2000). These results draw attention to the role of valence in emotional memory enhancement. It seems that the encoding of valenced but non-arousing stimuli is highly elaborative (for an overview, see Kensinger, 2004). Accordingly, findings of neuroimaging studies showed that emotional memory enhancement is associated with differential engagement of prefrontal networks that are important in controlled/elaborative encoding (Kensinger & Corkin, 2004). Moreover, negative memories contain more sensory details and are associated with the increased engagement of sensory processes during both encoding and retrieval (see Bowen, Kark, & Kensinger, 2018).

*Second*, it seems that memory is not better for all aspects of emotional stimuli. Central aspects of a (complex) stimulus tend to be better remembered, whereas peripheral (typically non-emotional) details are not (Easterbrook, 1959; Burke, Heuer, & Reisberg, 1992). Furthermore, a loss of contextual details can be detected for emotional stimuli despite enhanced subjective recollective experience during retrieval (Rimmele et al., 2011). While the visual details of a stimulus are more likely to be forgotten, memory is better for emotional information in tasks assessing “gist” memory (e.g. Adolphs, Denburg, & Tranel, 2001) that refers to memory for a broader, general content that includes connections among items with similar features. In fact, memory for the (visual) details of a stimulus and gist memory are strongly related to specific computational mechanisms in the hippocampus. We discuss these processes in the next subsection.

### Pattern separation and lure discrimination

One crucial feature of episodic memory is the ability to represent unique events from someone’s personal past (Tulving, 1972, 2002). This feature of episodic memory is strongly related to the process called pattern separation that is responsible for storing similar representations in distinct forms (Yassa & Stark, 2011). In other words, pattern separation reduces interference among similar inputs resulting in non-overlapping, unique memory representations.

At a neural level, pattern separation is a computational mechanism that refers to the separation of (partially) overlapping patterns of activation (see e.g. Gilbert & Kesner, 2006; Hunsaker & Kesner, 2013). Consequently, at a behavioral level, individuals become able to discriminate between (overlapping, similar) items (Kirwan & Stark, 2007; Yassa & Stark, 2011). In other words, as a result of pattern separation, the neuronal activities of brain circuits become distinct for two or more stimuli that share similar features. Specific subregions of the hippocampus are thought to play key roles in this process. In the past few decades, several computational models were developed to describe how the hippocampus supports interference resolution among similar sensory inputs enabling the formation of distinct memory representations (see Levy, 1989; McClelland, McNaughton, & O'Reilly, 1995; Treves & Rolls, 1994). In line with these models, there has been a growing body of evidence that specific subregions of the hippocampus, including the dentate gyrus (DG) and the CA3, perform domain-general pattern separation on overlapping sensory inputs (e.g. Kirwan et al., 2012; Yassa et al., 2011). For example, the DG is sensitive to relatively small changes in input (see Leutgeb, Leutgeb, Moser, & Moser, 2007; see also Yassa & Stark, 2011) and is therefore able to orthogonalize representations despite the extensive overlap between the features of different items. Hunsaker and Kesner (2013) argue that this process must occur during encoding and not at retrieval, however, the retrieval of unique (distinct) memories strongly depends on the successful separation of representations. Since non-interfering, non-overlapping memories are better remembered (Müller & Pilzecker, 1900; Underwood, 1957; Underwood & Postman, 1960), a causal relationship can be assumed between pattern separation and long-term memory retention.

In humans, the behavioral manifestation of putative neural mechanisms enabling pattern separation is usually assessed by the Mnemonic Similarity Task (MST; see e.g. Stark, Yassa, Lacy, & Stark, 2013). In this task, participants are presented with images of objects usually in an incidental encoding situation. The encoding phase is followed by a recognition test (typically with no delay between them) when subjects are shown old items (pictures they have seen at encoding [targets]) and completely new items (foils). Crucially, subjects are also presented with images that are visually similar but not identical to ones they have seen in the encoding phase of the task (lures). Typically, on the recognition test, participants have three response options: old, new, and similar. Subjects are required to decide whether they saw the image before, or not, or just see a similar picture to one they were presented with in the encoding phase of the task.

Studies investigated the behavioral manifestation of pattern separation (in humans) preferred to use some variation of the MST (for a recent overview, see Stark, Kirwan, & Stark, 2019) with the critical dependent variable of the so-called lure discrimination index, i.e. the difference between the rate of similar responses given to the lures items and the rate of similar responses given to the foils. Use of this index is suggested instead of a standard recognition memory score (i.e. old responses given to the target items minus old responses given to the foils) as a measure of hippocampal integrity (Stark et al., 2013), because this index seems to be more sensitive to hippocampal dysfunctions in pathological and normal ageing (Stark et al., 2013; Stark, Stevenson, Wu, Rutledge, & Stark, 2015) as well as in various psychiatric and neurological disorders (e.g. Hanert, Pedersen, & Bartsch, 2019; Kirwan et al., 2012). Importantly, although pattern separation is a computational process, lure discrimination reflects behavioral performance on a memory test where for correct discrimination, interference resolution is needed. In other words, these two concepts are descriptions of different levels of explanation. While pattern separation is a computational mechanism, lure discrimination is a behavioral construct, and this is the reason why authors prefer to use the term “Lure Discrimination Index” instead of “Pattern Separation Score” recently.

However, this index as a measure of successful lure discrimination is not the only one. Actually, from a behavioral perspective, the correct rejection of a stimulus that is similar to but not the same as a target (studied) item is assumed to be the manifestation of successful interference resolution (see e.g. Bakker, Kirwan, Miller, & Stark, 2008). Therefore, some studies prefer to use only old and new (and not similar) response options (e.g. Berron et al., 2018; Leal, Tighe, Jones, & Yassa, 2014; Szőllősi, Bencze, & Racsmány, 2020) (for the empirical investigation of different test variants with two and three response options, see Stark et al., 2015). In this variant of the MST lure rejections (i.e. the ratio of new responses given to the lure items minus the ratio of new responses given to the targets when response bias is controlled for) is used as a measure of lure discrimination. In sum, the principal idea is that only old responses given to the lures reflect incorrect lure discrimination, because in this case one incorrectly accepts the lure item as having been studied.

### Emotion, arousal, and pattern separation

Recently, it was suggested that increased arousal level plays a key role in superior discrimination between items with similar features and that superior discrimination as well as the reduction of interference effects leads to better memory retention (Segal, Stark, Kattan, Stark, & Yassa, 2012). Segal et al. (2012) used the MST to examine the relationship between arousal and lure discrimination. Participants were exposed to negative, arousing images selected from the International Affective Picture Set, and then they completed the MST with three response options (old, new, and similar). Higher noradrenergic activity, as indicated by elevated salivary alpha-amylase levels, was associated with higher lure discrimination score. The authors suggest that increased noradrenergic activity may modulate the involvement of DG in pattern separation via noradrenergic projections from the locus coeruleus and glutamatergic projections from the basolateral amygdala (see also McGaugh, 2000).

Another study investigated memory for emotional items and their visual details (Kensinger, Garoff-Eaton, & Schacter, 2006). The authors used a task that was very similar to the MST. First, they also used photographs of objects as stimuli. Second, participants were presented with not only old and new stimuli on the recognition test, but they saw visually similar items to ones they had been presented with at encoding. Participants had three response options (old, new, and similar) is this study as well. While there was no emotion effect for the similar items (i.e. no difference in the rate of similar responses given to the similar items, a measure that is equivalent to the lure discrimination index), the rate of old responses given to the old items was higher in the negative, than it was in the neutral condition. Based on this latter finding, the authors concluded that individuals tend to better remember the specific (visual) details of arousing stimuli when compared to memory for neutral information. It was also shown that the right amygdala was activated when specific visual details of emotional memories were accessed (Kensinger, Garoff-Eaton, & Schacter, 2007).

Another study also examined lure discrimination for emotionally arousing stimuli (Leal, Tighe, & Yassa, 2014). Subjects saw negative, positive, and neutral images, followed by a surprise recognition test, where there were old, new, and similar items in each condition, however, there were only two response options (old and new). The authors determined a lure rejection score as an index of lure discrimination by calculating the difference between the rate of new responses given to the similar stimuli and the rate of new responses given to the old stimuli. Interestingly, this lure rejection index was higher in the neutral than it was in the negative and positive conditions. Later, these behavioral findings were replicated, and an increased hippocampal DG/CA3 activity was found during lure correct rejections for the negative stimuli when compared to the neutral condition, while the amygdala responded to emotional stimuli regardless of the accuracy of lure discrimination (Leal, Tighe, Jones, & Yassa, 2014; for similar results, see Leal, Noche, Murray, & Yassa, 2017).

### Study objectives

In sum, studies of the relationship between lure discrimination, emotions, and arousal led to contradictory results. Therefore, we designed a study by combining the paradigms described above. We used the task developed by Leal et al. (Leal, Tighe, & Yassa, 2014), however, we made a crucial modification compared to the original paradigm. While in the original experiment, traditional old-new response options were used, there were three response options (old, new, and similar) in our study. The rationale for using an additional similar response option was to make a distinction between the forgetting of the original studied item (which might occur when one gives a new response to a lure) and the correct detection of similarities between the original studied item and its lure (which might occur when one gives a similar response to a lure) (see Kirwan & Stark, 2007). Although Kensinger et al. (2006) also used old-new-similar response options, the authors used only negative and neutral (but not positive) stimuli. Our paradigm together with the methodological modifications allowed us to analyze various measures. We calculated a standard recognition memory score (rate of old responses given to the old items minus the rate of old responses given to the new items) and a lure discrimination index (rate of similar responses given to the lure items minus the rate of similar responses given to the foil items) following the tradition of several previous studies (e.g. Kirwan et al., 2012; Segal et al., 2012; Stark et al., 2013). Additionally, we analyzed the ratio of new responses given to the lure items minus the ratio of new responses given to the targets, as Leal and colleagues (Leal, Tighe, Jones, & Yassa, 2014) did. The rationale for analyzing this measure was to examine whether we would find a similar pattern of results despite the methodological modifications we made.

We assumed that better memory for emotionally arousing study materials is due to more distinct representations of emotional memories (see also Schmidt & Saari, 2007; Talmi, 2013). Since pattern separation is the process which minimizes interference between memory representations with similar features (Yassa & Stark, 2011), and noradrenergic activity is supposed to facilitate interference resolution (Levens, Devinsky, & Phelps, 2011; Levens & Phelps, 2008), we hypothesized higher lure discrimination score for the emotionally arousing stimuli compared to the neutral, non-arousing study material. We hypothesized that arousal and not the valence of the stimuli affects interference resolution. Therefore, as a novelty, we aimed to separate the role of arousal and the role of valence in lure discrimination. We analyzed the data on the basis of the arousal level of the stimuli by contrasting lure discrimination scores between the low and high arousing stimuli within the negative and the positive condition, separately. Importantly, since pattern separation is suggested to be occur at the time of encoding (Hunsaker & Kesner, 2013), we believed that our study would help to better understand the nature of encoding of emotionally arousing information. We assumed that the elaborative encoding of arousing information provides a basis for superior interference resolution.

## Materials and methods

### Participants

Altogether 104 undergraduate students participated in the study. Twenty-two subjects were asked to rate the emotional valence of the stimuli (6 men; age: 19–26 years, *M* = 22.1, *SD* = 1.9), and another group of 22 subjects (3 men; age: 20–31 years, *M* = 22.1, *SD* = 2.4) were instructed to rate the arousal level of the stimuli.

Required sample size was calculated on the basis of a pilot study where participants (*n* = 20) completed the memory task. We aimed at analyzing standard recognition performance, lure discrimination, and the ratio of new responses for the lures (minus the ratio of new responses for the targets). We found the smallest (non-significant) effect for standard recognition performance with an effect size of η_p_^2^ = 0.05. Based on this effect size value, we used G*Power (Version 3.1.9.2; see Faul, Erdfelder, Lang, & Buchner, 2007) to calculate required sample size (test family: *F*-test; statistical test: repeated measures analysis of variance [ANOVA]) with an alpha error probability of 0.05, with three levels (as the number of conditions: negative, neutral, and positive stimuli), and a power of 0.95. Based on the output parameters, required sample size was a minimum of *n* = 51. Expecting some drop out, we collected data from 60 participants. Two subjects were excluded from the sample, because they did not complete the whole task. Therefore, we analyzed the data of 58 participants (12 men; age: 19–28 years, *M* = 21.5, *SD* = 1.8).

Participants had no history of psychiatric or neurological disorders and had normal or corrected-to-normal vision. All subjects received extra course credit for participation and gave written informed consent. The study was approved by the United Ethical Review Committee for Research in Psychology, Hungary. The study has been carried out in accordance with The Code of Ethics of the World Medical Association (Declaration of Helsinki) for experiments involving humans.

### Stimuli and stimulus validation

In the encoding phase of the emotional MST, subjects were presented with 156 pictures (52 negative, 52 neutral, and 52 positive). We used the stimulus set of Leal et al. (2014). Stimuli were color photographs of scenes. The original version of the MST (Stark et al., 2013) uses photographs of everyday objects on white backgrounds as stimuli and not scenes. Importantly, these two stimulus types (objects and scenes) show strong similarities (e.g. both stimulus types are sensitive to age-related changes in lure discrimination performance), and lure discrimination performances in different test variants with objects and scenes as stimuli strongly correlate (Stark & Stark, 2017).

Both in the original (Yassa et al., 2011; see also Kirwan & Stark, 2007) and the emotional version of the MST (Leal, Tighe, & Yassa, 2014) a mnemonic similarity measure was used to normalize the stimulus sets. Mnemonic similarity is based on the ratio of false alarm rates for the lures (the probability of responding old to a lure item). Leal and colleagues reported no differences in false alarm rates between the conditions (negative, neutral, and positive). The authors also collected similarity ratings by involving an independent group of participants and reported no differences between the negative, neutral, and positive items. We used this stimulus set in our study to make our results and the findings of this previous study more comparable.

In our study, two independent groups of subjects rated either the emotional valence (where 1 = *negative*, 9 = *positive*) or the arousal level (where 1 = *least arousing*, 9 = *most arousing*) of the images on nine-point scales. Participants were presented with the stimuli in a random order, and each picture remained in the middle of the computer screen for 2500 ms (with an inter-stimulus interval [ISI] of 500 ms). Subjects were asked to respond by using a standard keyboard of a computer, and the response options (i.e. the endpoints of the scale) were presented in the bottom of the computer screen during the whole task.

Based on the results of stimulus validation we classified each emotional stimulus into one of two categories (low arousing and high arousing) within the negative and the positive condition, separately. Within both conditions (positive and negative), there were 26 images. Half of the items were classified into the low arousing stimulus set, whereas the remaining images were classified into the high arousing stimulus set. Therefore, the categories were as follows: low arousing negative (13 stimuli), high arousing negative (13 stimuli), low arousing positive (13 stimuli), and high arousing positive (13 stimuli).

### The emotional mnemonic similarity task: Experimental design and procedure

**Figure 1.**
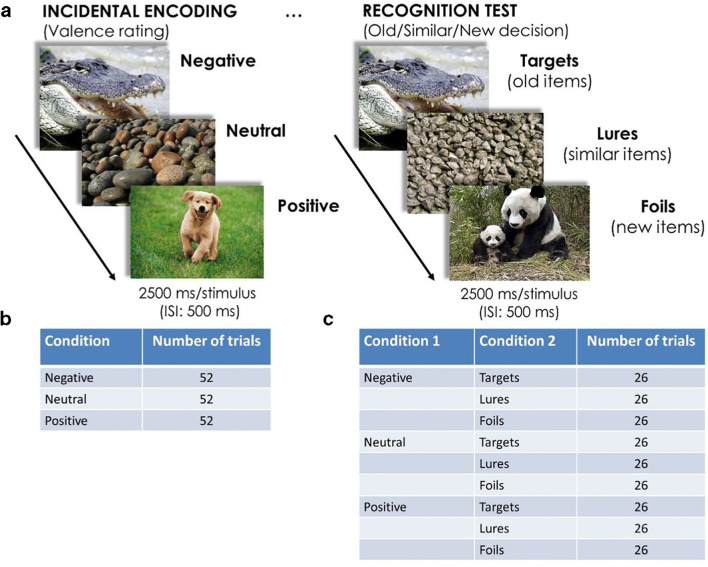
(A) The procedure of the emotional Mnemonic Similarity Task. Experimental conditions in the (B) encoding and (C) test phases of the task. *Note(s).* ISI = inter-stimulus interval.

The task consisted of two phases with no delay between them, an incidental encoding phase and a recognition test. The procedure and the experimental design are illustrated in Fig. 1.

In the *incidental encoding phase*, subjects were shown the 156 pictures in a random order, and their task was the same as participants’ task was during stimulus validation (i.e. when an independent group of subjects rated the emotional valence of the stimuli), see Fig. 1a and Fig. 1b. The encoding phase was preceded by 10 practice trials with 4 negative, 2 neutral, and 4 positive pictures. Practice trials were presented in a random order.

The encoding phase was immediately followed by a surprise *recognition test* containing 234 trials. In the test phase, a 3 × 3 experimental design was used (see Fig. 1a and Fig. 1c). Subjects were shown negative, neutral, and positive pictures; within each condition there were target, lure, and foil items. Targets (26 negative, 26 neutral, and 26 positive images) were old items that were presented in the encoding phase. Lure items (26 negative, 26 neutral, and 26 positive images) were similar but not identical to ones that were presented at encoding. Foils (26 negative, 26 neutral, and 26 positive images) were not presented at all in the encoding phase of the task. Each picture remained in the middle of the computer screen for 2500 ms with a 500-ms ISI. Stimuli were presented in a different random order for each participant.

Participants were required to give an “old” response to repetitions (to stimuli they saw in the encoding phase [targets]), and to give a “new” response to completely new pictures (to stimuli they have not seen before [foils]). Crucially, subjects were instructed to give a “similar” response to pictures that were similar but not identical to ones they saw in the encoding phase (lures). Response options (F = *old*, H = *similar*, and K = *new*) remained in the bottom of the computer screen during the whole recognition test.

### Data analysis

No published or in press manuscripts use(d) the same dataset. We used an alpha level of *p* < .05 for all statistical tests. We report partial eta-squared (η^2^_p_) value as a measure of effect size for ANOVAs and Cohen’s *d* value as a measure of effect size for *t*-tests.

#### Valence and arousal ratings

For valence and arousal ratings that were given during stimulus validation repeated measures ANOVAs were conducted with three levels (negative, neutral, and positive). During post hoc analyses, simple contrasts were used for pairwise comparisons.

As for the data of stimulus validation, valence ratings that were given during encoding were compared between the conditions with a repeated measures ANOVA with three levels (negative, neutral, and positive) followed by simple contrast analyses for pairwise comparisons. Additionally, we compared valence ratings that were given during stimulus validation and valence ratings that were given in the encoding phase of the memory task. Therefore, we conducted a 2 × 3 mixed-design ANOVA with Phase (Stimulus validation and Encoding) as a between-subjects factor and Valence (Negative, Neutral, and Positive) as a within-subjects variable.

#### Memory performance

*Standard recognition score* was determined by calculating the difference between the rate of old responses given to the targets and the rate of old responses given to the foils (Old | Targets – Old | Foils). Additionally, and importantly, a *lure discrimination index* (see e.g. Stark et al., 2013) was determined by calculating the difference between the rate of similar responses given to the lures and the rate of similar responses given to the foils (Similar | Lures – Similar | Foils). Finally, we analyzed the ratio of new responses given to the lures minus the ratio of new responses given to the targets (New | Lures – New | Targets) as Leal and colleagues (Leal, Tighe, Jones, & Yassa, 2014) did in their experiment.

For each score, repeated measures ANOVAs were conducted with the negative, neutral, and positive conditions as three levels. As post hoc analyses, a list of simple contrasts was conducted for pairwise comparisons (negative vs. neutral, neutral vs. positive, and negative vs. positive).

#### The role of arousal and valence

We conducted paired samples *t*-tests to compare valence and arousal ratings between the low and high arousing stimuli in the two conditions (negative and positive) separately. When we analyzed memory performance on the basis of the arousal level of the stimuli, we did not calculate a standard recognition memory score and we did not calculate a lure discrimination index. Specifically, response bias was not controlled for. There were negative, neutral, and positive foils in our study, but we did not conduct data on the arousal level of the foils. Therefore, we did not subtract old responses given to the foils from old responses given to the targets. Due to the same reason, we did not subtract similar responses given to the foils from similar responses given to the lures. Instead, we analyzed the ratio of old responses for the target items and the ratio of similar responses for the lure items. These are alternative measures of recognition memory performance and lure discrimination, respectively (see Stark et al., 2019), and are frequently used (e.g. Holden, Toner, Pirogovsky, Kirwan, & Gilbert, 2013; Toner et al., 2009), but do not account for overall tendency of responding either old or similar. We used paired samples *t*-tests to compare old responses given to the targets between the low and high arousing stimuli in the positive and negative conditions, separately. We conducted the same analyses for similar responses given to the lures.

## Results

### Stimulus validation: Valence and arousal ratings

**Fig. 2:**
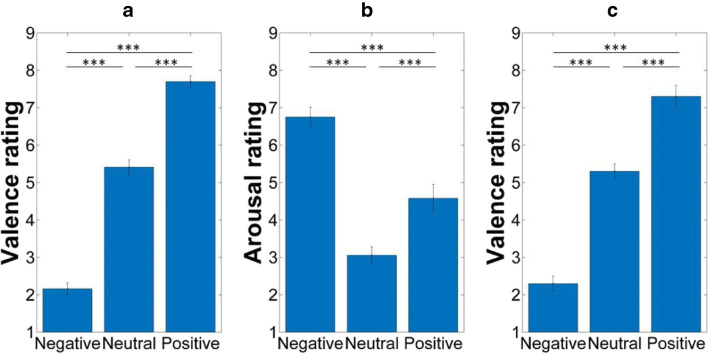
(A, C) Emotional valence and (B) arousal level of the stimuli. *Note(s).* Data presented in Fig. 2a and data presented in Fig. 2b are based on the results of stimulus validation (when two independent groups of subjects rated the emotional valence and the arousal level of the stimulus set); data presented in Fig. 2c is based on the results of the encoding phase of the memory task. ^***^p < .001. Error bars represent the standard errors of the means

The ANOVAs indicated significant differences between the conditions for both valence, *F*(2, 42) = 217.13, *p* < .001, η^2^_p_ = 0.91, and arousal, *F*(2, 42) = 46.61, *p* < .001, η^2^_p_ = 0.69 (see Fig. 2a and 2b, respectively). Compared to the neutral stimuli, participants gave lower valence ratings for the negative pictures, *F*(1, 21) = 91.33, *p* < .001, η^2^_p_ = 0.81, and they gave higher valence ratings for the positive pictures, *F*(1, 21) = 511.28, *p* < .001, η^2^_p_ = 0.96. Positive and negative images also differed in valence ratings, *F*(1, 21) = 347.31, *p* < .001, η^2^_p_ = 0.64.

Positive, *F*(1, 21) = 18.41, *p* < .001, η^2^_p_ = 0.47, and negative images, *F*(1, 21) = 143.37, *p* < .001, η^2^_p_ = 0.87, were more arousing than the neutral stimuli. However, and importantly, the negative images were more arousing than the positive ones, *F*(1, 21) = 21.19, *p* < .001, η^2^_p_ = 0.50. This pattern of findings is in line with the results of Leal et al. (2014) who also found that the negative images were more arousing as compared to the positive (and neutral) stimuli. Also, a couple of previous studies used stimulus sets where the negative images were more arousing than the positive stimuli (e.g. Garavan, Pendergrass, Ross, Stein, & Risinger, 2001). This difference in the arousal level of the positive and negative images led to the decision of comparing memory performances for the low and high arousing stimuli within the conditions (positive and negative) separately.

### The emotional mnemonic similarity task

#### Encoding phase: Valence rating

We found a similar pattern of results as we did during stimulus validation (see Fig. 2c): valence ratings differed between the conditions, *F*(2, 114) = 623.22, *p* < .001, η^2^_p_ = 0.92. Compared to the neutral stimuli, the positive pictures were given higher ratings, *F*(1, 57) = 515.12, *p* < .001, η^2^_p_ = 0.90, and the negative pictures were given lower ratings, *F*(1, 57) = 548.54, *p* < .001, η^2^_p_ = 0.91. The negative and the positive images also differed, *F*(1, 57) = 688.40, *p* < .001, η^2^_p_ = 0.93.

Additionally, we compared valence ratings that were given during stimulus validation and valence ratings that were given in the encoding phase of the memory task. As expected, Valence had a main effect on the ratings, *F*(2, 156) = 711.18, *p* < .001, η^2^_p_ = 0.90, as ratings differed between the conditions (negative, neutral, and positive) both during stimulus validation and in the encoding phase of the memory task. More importantly, Phase had no main effect on the ratings, *F*(1, 78) = 0.44, *p* = .51, η^2^_p_ = 0.01, and the Phase x Valence interaction was also not significant, *F*(2, 156) = 2.75, *p* = .10, η^2^_p_ = 0.04. In brief, the results of the encoding phase in the MST replicated the findings of stimulus validation.

#### Memory performance

**Fig. 3:**
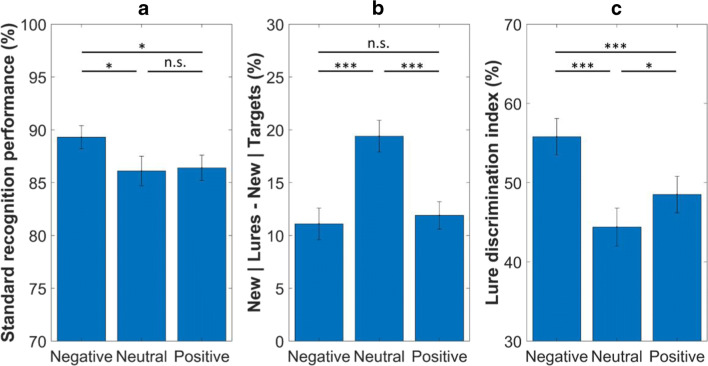
Recognition performance in the emotional Mnemonic Similarity Task. *Note(s).* (A) Old | Targets – Old | Foils. (B) New | Lures – New | Targets. (C) Similar | Lures – Similar | Foils ^*^p < .05, ^***^p < .001, *n.s.* = non-significant. Error bars represent the standard errors of the means

For standard recognition performance (Fig. 3a) we found a significant difference between the conditions, *F*(2, 114) = 3.95, *p* = .02, η^2^_p_ = 0.07. Participants showed better recognition performance for the negative pictures than they did for the neutral, *F*(1, 57) = 5.73, *p* = .02, η^2^_p_ = 0.09, and positive stimuli, *F*(1, 57) = 5.51, *p* = .02, η^2^_p_ = 0.09. There was no significant difference between the neutral and positive conditions, *F*(1, 57) = 0.08, *p* = .78, η^2^_p_ < 0.01.

New responses given to the lures (minus new responses given to the targets) also differed between the conditions, *F*(2, 114) = 19.30, *p* < .001, η^2^_p_ = 0.25; see Fig. 3b. This score for the neutral items was higher than it was for the negative, *F*(1, 57) = 29.09, *p* < .001, η^2^_p_ = 0.34, and positive stimuli, *F*(1, 57) = 25.26, *p* < .001, η^2^_p_ = 0.31. There was no significant difference between the negative and positive conditions, *F*(1, 57) = 0.38, *p* = .54, η^2^_p_ < 0.01.

Finally and most importantly, for the lure discrimination index (Fig. 3c), we found significant differences between the conditions as well, *F*(2, 114) = 20.88, *p* < .001, η^2^_p_ = 0.27. The lure discrimination value was higher in the negative condition than it was in the neutral, *F*(1, 57) = 37.78, *p* < .001, η^2^_p_ = 0.40, and positive conditions, *F*(1, 57) = 17.56, *p* < .001, η^2^_p_ = 0.24. Additionally, we found higher lure discrimination value for the positive pictures than we did for the neutral ones, *F*(1, 57) = 5.48, *p* = .02, η^2^_p_ = 0.09.

These latter results reflect differences in the arousal level of the stimuli, as the negative pictures were more arousing than the positive stimuli. However, to make a clear conclusion whether lure discrimination performance is modulated by arousal or valence, we conducted an additional analysis.

#### The role of arousal and valence

We classified each stimulus into one of the following categories: low arousing negative, high arousing negative, low arousing positive, and high arousing positive (for arousal and valence ratings per category, see Table 1). Arousal ratings (that were given during stimulus validation) differed significantly between the low and high arousing stimuli in both the negative, *t*(21) = 8.11, *p* < .001, *d* = 1.73, and positive conditions, *t*(21) = 6.61, *p* < .001, *d* = 1.41. On the other hand, there was no significant difference between them in their valence ratings (that were also given during stimulus validation), negative: *t*(21) = 0.65, *p* = .73, *d* = 0.08, positive: *t*(21) = 0.79, *p* = .44, *d* = 0.17.

**Table 1:** Classification of the stimulus set on the basis of arousal: Arousal and valence ratings (the scales ranged between 1 and 9)

**Rating**	**Negative stimuli**			**Positive stimuli**	
	**Low arousal**	**High arousal**		**Low arousal**	**High arousal**
Arousal	5.85 (0.31)	7.47 (0.24)		3.75 (0.37)	5.51 (0.42)
Valence	2.32 (0.18)	2.29 (0.22)		7.69 (0.18)	7.60 (0.15)

*Note(s).* Data presented in the Table is based on the results of stimulus validation (when two independent groups of subjects rated the emotional valence and the arousal level of the stimulus set). Values represent the means; standard errors of the means are shown in parentheses

**Fig. 4:**
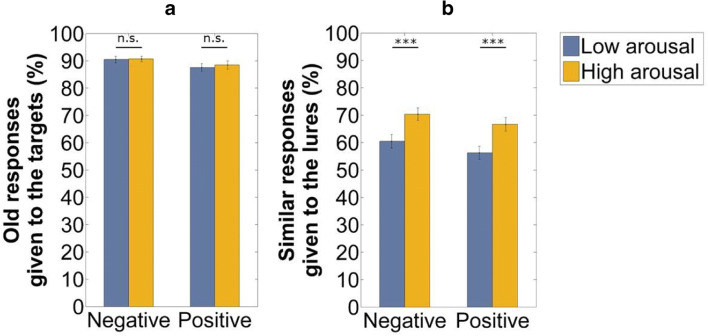
Comparison between the low and high arousing stimuli: (A) old responses given to the targets as a measure of recognition memory performance and (B) similar responses given to the lures as a measure of lure discrimination. *Note(s). n.s.* = non-significant, ^***^p < .001. Error bars represent the standard errors of the means

There was no significant difference in the ratios of old responses given to the targets between the low and high arousing items, negative: *t*(57) = 0.28, *p* = .78, *d* = 0.04, positive: *t*(57) = 0.52, *p* = .61, *d* = 0.07 (see Fig. 4a). In contrast, arousal had a beneficial effect on the ratio of similar responses given to the lures for the negative stimuli, *t*(57) = 4.01, *p* < .001, *d* = 0.53, and also for the positive stimuli, *t*(57) = 4.71, *p* < .001, *d* = 0.62 (see Fig. 4b).

## Discussion

The aim of the present study was to investigate lure discrimination for emotionally arousing memories with new behavioral methodologies, and we found differences in memory performance for the neutral and emotional stimuli according to various measures. These immediate effects support the notion that emotions do affect encoding, and not only consolidation (see also Chainay et al., 2012; Dewhurst & Parry, 2000; Hamann, 2001; Talmi & McGarry, 2012).

### Standard recognition memory performance

First, subjects showed better standard recognition memory performance (as measured by the ratio of old responses given to the targets minus old responses given to the foils) for the negative items, when compared to the neutral and positive stimuli. This finding is not only a replication but also an extension of Kensinger et al. (2006), as we used positive and not only negative and neutral materials. Results of our post hoc analysis suggest that this memory enhancement was modulated by valence, as standard recognition performance did not differ between the low and high arousing stimuli either in the negative or in the positive condition. Furthermore, we found no difference between the neutral and positive items despite differences in their arousal levels. These findings are in line with previous results showing that valence (and not only the arousal level of the stimuli) plays a key role in emotional memory enhancement (Kensinger & Corkin, 2003; Ochsner, 2000). It seems that negative items are better remembered when compared to memory for positive materials (Ochsner, 2000). This effect might be due to the elaborative encoding of emotional materials (see Bowen et al., 2018; Kensinger, 2004). Accordingly, it has also been demonstrated that memories for negative events contain more sensory information (see Bowen et al., 2018), and recognition memory performance might benefit from these detailed representations. It should be also noted, that the small magnitude of this effect (cf. a maximum difference of 3% in recognition rates between the conditions in the present study) together with relatively low sample sizes in some former studies might play important roles in null effects on immediate memory tests reported previously (for a review, see Hamann, 2001).

On the other hand, according to the result of stimulus validation, negative stimuli were given higher arousal ratings as compared to the positive items. This result together with the findings that standard recognition memory scores did not differ between the low and high arousing negative items as well as between the low and high arousing positive items might indicate that there is a minimum level of arousal that affect recognition memory performance. This suggestion is in line with the result of Canli, Zhao, Brewer, Gabrieli, and Cahill (2000) who showed that recognition memory for pictures of scenes was enhanced only for those emotional stimuli that were associated with a high level of arousal. The authors suggested that there is a threshold of arousal below which memory is not enhanced. Nevertheless, we believe that not the arousal level of the stimuli affected memory performance in our study, however, future studies are needed to clarify this issue.

### Lure discrimination

Our results showed that lure discrimination for the emotional items was better than it was for the neutral stimuli. Moreover, subjects showed superior lure discrimination performance for the negative items, when compared to the positive stimuli. This pattern of findings reflects differences in the arousal level of the stimuli, as the negative pictures were more arousing than the positive images. Results of our follow-up analysis suggest that superior lure discrimination for the emotional stimuli was modulated by arousal (and not by valence), as we found higher lure discrimination scores for the arousing stimuli. Specifically, the high arousing negative stimuli were better separated in comparison with the low arousing negative stimuli, and a similar pattern of results was found for the positive items.

One important issue is why lure discrimination is modulated by arousal, while standard recognition performance might be not. Lure discrimination refers to the correct discrimination between targets and critical lures, whereas standard recognition score reflects the discrimination between the target items and foils (see Bakker et al., 2008). Since there is a high overlap between the (visual) features of targets and lures, for correct discrimination between these types of stimuli, interference resolution is needed. In the absence of overlap between the representations of targets and foils, there is no such interference effects. Several previous studies reported a dissociation between lure discrimination and standard recognition performance. For example, while pathological and healthy ageing affects pattern separation performance (due to dysfunctional interference resolution), it has no impact on performance when only target-foil discrimination is needed (e.g. Stark, Yassa, & Stark, 2010; Stark et al., 2013). We suggest that arousal has a beneficial effect on the reduction of interference effects (see also Levens et al., 2011; Levens & Phelps, 2008), and as a consequence, it selectively facilitates lure discrimination performance.

Although we analyzed behavioral data, our findings together with results of previous neuroimaging studies suggest that increased noradrenergic activity promotes pattern separation. Since a high density of noradrenergic receptors can be found in the DG (Harley, 2007), it seems plausible that arousal modulates pattern separation via noradrenergic projections to the DG (see also Segal et al., 2012). Other authors also suggest that NA projections from the locus coeruleus to the hippocampus play a role in neural pattern separation (Leal & Yassa, 2015; Yassa & Stark, 2011). Importantly, pattern separation is suggested to be fast that occurs when one encounters with a stimulus (e.g., Bakker et al., 2008; see also Hunsaker & Kesner, 2013). Therefore, if pattern separation occurs at the time of encoding and noradrenergic activity is assumed to facilitate this process then noradrenergic modulation must also occur at encoding. Future studies are needed, however, to test this hypothesis. Several studies have demonstrated that stress hormones, including high noradrenaline levels, have beneficial effects on memory encoding and memory consolidation processes (for an overview, see Roozendaal & Hermans, 2017). It has been also shown that arousal facilitates interference resolution (Levens et al., 2011; Levens & Phelps, 2008), and that memory benefits from the distinctiveness of arousing experiences (Schmidt & Saari, 2007; Talmi, 2013). Our findings together with these previous results suggest that arousal (and probably high noradrenergic activity) promotes processes that are responsible for reducing interference between similar, overlapping memory representations that might play a crucial role in better memory for emotional experiences.

Contrary to our results, a previous study (Kensinger et al., 2006) found no emotion effect on the ratio of similar responses given to the similar (lure) items, which measure is identical to the lure discrimination index we used. This different pattern of results might be explained by methodological differences between the studies. In this previous study, stimuli were presented for either 250, 500, or 1000 ms, whereas presentation duration was 2500 ms in our study. We believe that longer exposure duration promotes the more elaborative encoding of emotional information. It seems especially important, because lure discrimination benefits from detailed memory representations. Accordingly, Kensinger and colleagues found enhanced recognition memory performance for the arousing stimuli when stimulus presentation duration was either 500 or 1000 ms and not when presentation duration was 250 ms. In agreement with the authors, we believe that the formation of detailed memory representations is more likely to occur when presentation duration is longer (2500 ms in our study) and even a relatively short time interval can make a difference. In other words, recognition memory for emotionally arousing materials benefits from longer stimulus presentation duration (Kensinger et al., 2006). For this reason, it is likely that even longer presentation duration is needed for the formation of even more detailed representations and for superior discrimination between arousing materials.

Finally, we replicated the findings of Leal and colleagues (Leal, Tighe, Jones, & Yassa, 2014) as the rate of new responses for the lures was higher for the neutral memories than it was for the emotional stimuli. The authors used this so-called lure rejection index as a measure of successful lure discrimination, and this value ranged between 40 and 70%. In our study, this value was relatively low (ranged from 11 to 19%) due to our methodological modification (i.e. an additional similar response option). The main point is here, however, that the same pattern of results was found in both studies. Specifically, the rate of new responses for the lures was higher for the neutral stimuli, as compared to the emotional items, whereas there was no reliable difference between the positive and negative conditions.

In contrast, when we assessed lure discrimination by calculating the rate of similar responses given to the lures (and not by lure rejections, as Leal and colleagues did), we found superior lure discrimination for the emotional stimuli. This finding therefore pointed out that different measures of lure discrimination (similar and new responses given to the lure items) might reflect different underlying processes. In fact, both indices have advantages and disadvantages. When participants have the opportunity to give similar responses (such as in our study), results strongly depend on how they interpret the instructions. As an advantage, when participants have only two response options the standard signal detection theory (see e.g. Lockhart & Murdock, 1970; Stanislaw & Todorov, 1999) can be used to assess performance by calculating discriminability between different stimulus types (e.g. Stark et al., 2015; Szőllősi et al., 2020; for an overview, see Stark et al., 2019). Another argument in favour of using fewer response options is the reduction of task difficulty that seems especially important when specific populations, such as older adults are involved (Berron et al., 2018). However, when participants have three response options, similar and new responses can be separated. As it was described in a previous paper (Kirwan & Stark, 2007, page 626) “one must allow not only old and new responses, but similar responses as well, so that one can assess whether, when subjects fail to respond old, they have merely forgotten the original item or whether they can retrieve a memory of the original item and know that the present one is similar to, but not the same as the original”. The main point is here that new responses given to the lures cannot be really (or cannot be always) considered as correct responses, as the participant might have not noticed the (visual) differences between the target item and its lure.

## Summary and conclusions

In sum, we argue that in the present study standard recognition memory performance was modulated by valence, whereas the discrimination between visually similar items was affected by the arousal level of the stimuli. We suggest that the arousal level of emotional stimuli (probably via increased noradrenergic activity) facilitates interference resolution among overlapping memory representations during encoding, and that this improvement in discrimination might play an important role in memory enhancement for emotional experiences.

## Data Availability

Datasets related to this article can be found at https://osf.io/drs7w/, an open-source online data repository (Open Science Framework).
